# Negative Pressure Pulmonary Edema Related to Laryngospasm and Upper Airway Obstruction in a Patient With Treacher Collins Syndrome

**DOI:** 10.7759/cureus.14426

**Published:** 2021-04-11

**Authors:** Jenny Zhao Cheng, Jingping Wang

**Affiliations:** 1 Anesthesia, Critical Care and Pain Medicine, Massachusetts General Hospital, Boston, USA; 2 Anesthesia, Harvard Medical School, Boston, USA

**Keywords:** laryngospasm, upper airway obstruction, negative pressure pulmonary edema, treacher-collins syndrome, difficult airway algorithm

## Abstract

Laryngospasm is an uncommon complication of anesthesia in adults but more common in pediatric anesthesia, which could present similarly to supraglottic upper airway obstruction. The management of such airway complications is even more difficult in patients with difficult mask ventilation and intubation. Our case illustrated the management of laryngospasm and negative pressure pulmonary edema in a patient with Treacher Collins syndrome. A literature search revealed few previous similar reports. We demonstrated an algorithm to differentiate between the true laryngospasm from the supraglottic upper airway obstruction, the management of laryngospasm in patients with difficult airways, and the recognition and management of negative pressure pulmonary edema as a complication of laryngospasm.

## Introduction

Laryngospasm is an uncommon yet potentially life-threatening complication of anesthesia in adults but more common in pediatric anesthesia. It is defined as the glottic closure due to a reflex constriction of the laryngeal muscles [1].­­ Laryngospasm often presents with high pitched inspiratory stridor during incomplete airway closure or silent chest movements with deteriorating oxygen saturation when the airway obstruction is complete. Both presentations could also be seen in the supraglottic upper airway obstruction. The conventional management of laryngospasm involves the use of continuous positive airway pressure (CPAP) to push open the vocal cords, low dose succinylcholine to relax the vocal cords, deepening the depth of anesthesia with intravenous anesthetics or the application of Larson Maneuver, which refers to the application of pressure on the styloid process behind the posterior ramus of the mandibles [2]. However, it becomes more complex when the patient is difficult to ventilate with a face mask and difficult to intubate. Negative pressure pulmonary edema (NPPE) is a potential complication of laryngospasm, which should be recognized early and treated promptly.

Our case illustrated a patient with Treacher Collins syndrome (TCS), who developed laryngospasm and subsequently NPPE. The literature search revealed few previous similar reports. We demonstrated an algorithm to differentiate between the true laryngospasm from the supraglottic upper airway obstruction, the management of laryngospasm in patients with difficult airways, and the recognition and management of NPPE as a complication of laryngospasm. Written informed consent was obtained from the patient’s legal guardian.

## Case presentation

**Figure 1:**
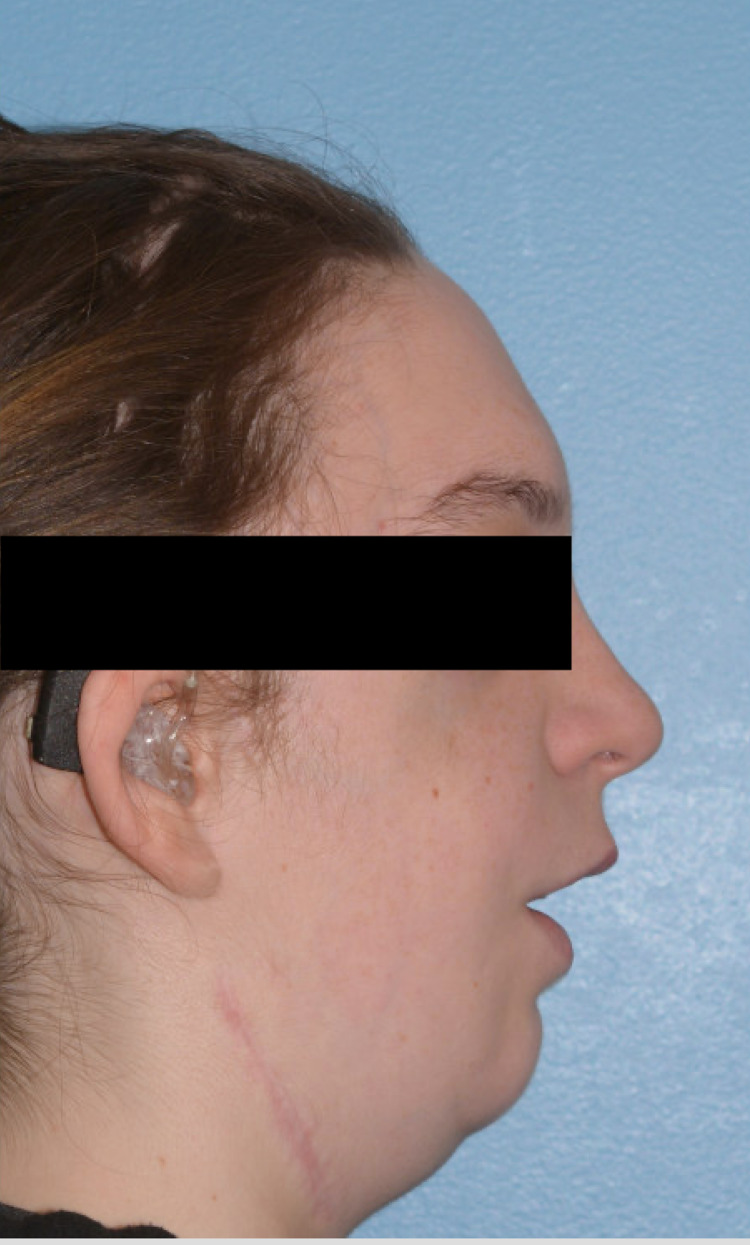
Lateral profile of the patient with Treacher Collins syndrome

A 16-year-old female (169cm, 70kg, BMI 24.5) with TCS status post genial tubercle advancement, mandibular reconstruction with graft, and LeFort I osteotomy and craniotomy, presented for removal of left maxillary bone plate and screws and implantation of customized right and left malar implants. Patient’s previous anesthesia record revealed that she had adequate mask ventilation with an oral airway, chin and jaw lift and that she was intubated electively with a nasal fiberoptic scope and extubated in the operating room the following day. Patient’s exam revealed Mallampati IV, short thyromental distance (3cm), small chin, limited mouth opening (2cm), and large tongue relative to oral cavity (Figure 1). General anesthesia with an elective asleep fiberoptic intubation was performed. After induction, mask ventilation was feasible with jaw and chin lift and the patient was intubated with 6.5 oral endotracheal tube with cuff via fiberoptic bronchoscope with one attempt.

The surgery was uneventful. During the operation, the patient received 100mcg of fentanyl at the induction and 0.5mg hydromorphone at the incision for pain control. Since incision, no more opioid was given to avoid the risk of respiratory depression at extubation. Prior to extubation, the surgeon inserted an orogastric tube to suction any bloody secretion to prevent the risk of aspiration. At the end of the 3.5-hour procedure, the neuromuscular blockade was reversed with neostigmine and glycopyrrolate with a Train-of-Four of four twitches and no fade. The patient was breathing spontaneously, following simple commands and opening her eyes. The decision was made to extubate her in the operating room. Soon after extubation, silent chest movement was observed with no end-tidal carbon dioxide (ETCO_2_) and oxygen saturation (SpO­_2_) deteriorated rapidly. An oral airway was inserted, and positive pressure bag-mask ventilation was initiated. The patient continued to have no ETCO_2_, then two nasal airways were inserted in the patient’s bilateral nostrils with chin lift, jaw lift by using a two-person ventilation technique, and 110mg of propofol was administered intravenously. At the same time, the emergent surgical airway was called to be ready in a case of an impossible re-intubation if laryngospasm persisted. Laryngospasm improved after propofol bolus as ETCO­­_2_ returned and SpO_2_ improved significantly to 100% within two minutes. The patient’s respiratory status eventually stabilized with oxygen saturation of 97%, breathing on her own with both oral and nasal airway adjuncts without jaw lift or chin lift support.

**Figure 2:**
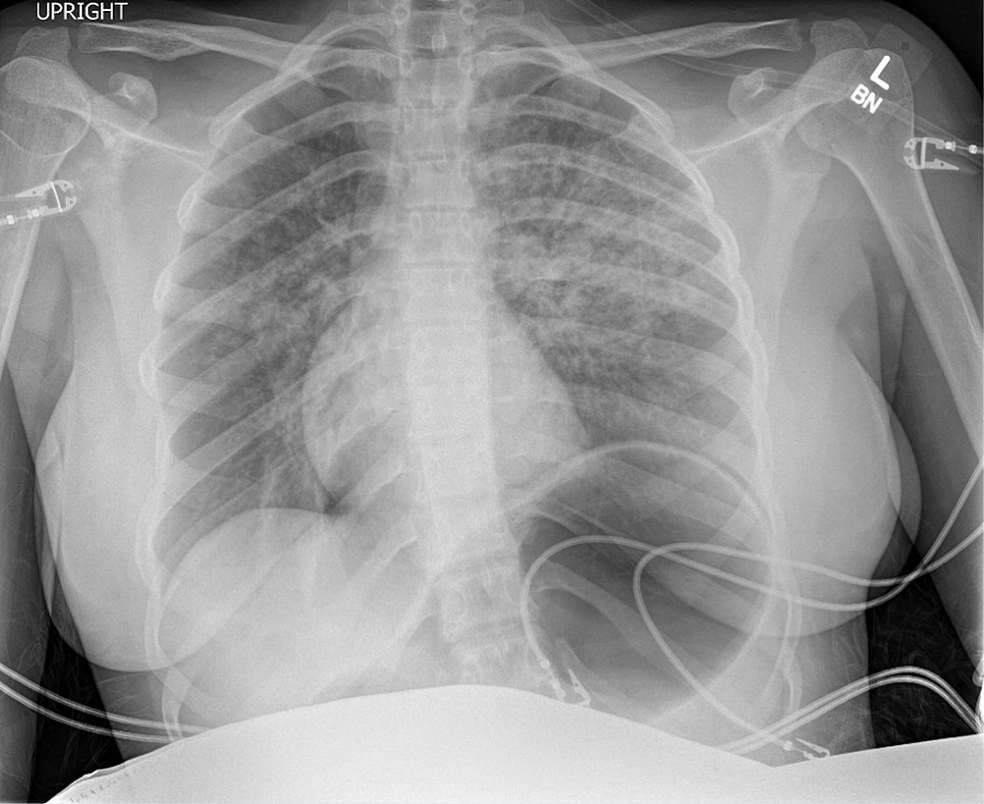
Anterior-posterior chest radiograph of the patient immediately after the surgery in the recovery unit.

**Figure 3:**
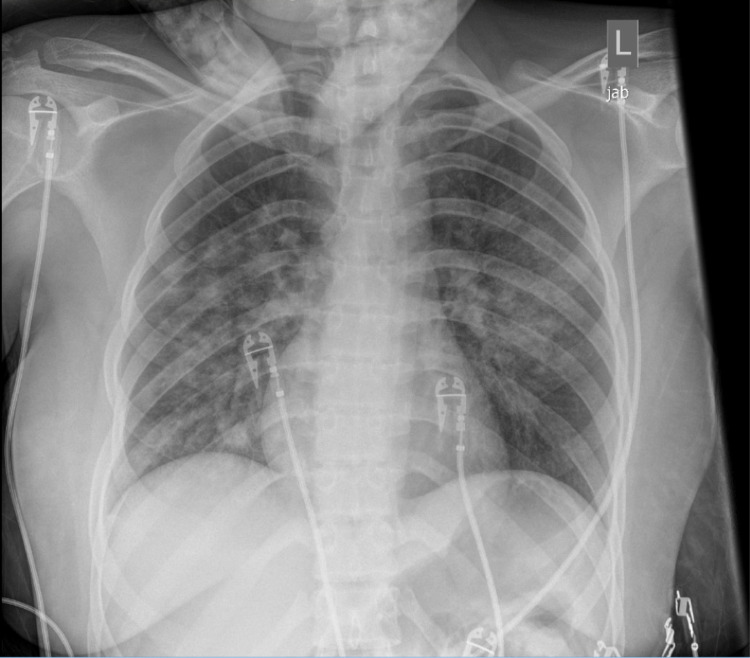
Anterior-posterior chest radiograph of the patient on post-operative day 1.

At the time patient was transferred to post-anesthesia recovery unit, she developed hypoxemia with oxygen saturation in the low 80s despite her good respiratory effort. The patient was awake, alert and sitting in bed. Non-rebreather mask with high flow supplemental oxygen was initiated. Physical exam revealed that her lungs were clear to auscultation bilaterally without wheezes or crackles. No pink frothy secretion was seen when the patient was asked to take deep breaths and cough. Bedside chest radiograph revealed bilateral pulmonary opacities and gastric distension (Figure 2), leading to the diagnosis of NPPE in the setting of her negative cardiac history. The gastric distension was likely from insufflation of the stomach secondary to the attempted positive pressure ventilation via face mask. The patient then received diuretics and was admitted to the intensive care unit for airway monitoring and management of pulmonary edema. On post-operative day 1, the patient improved clinically. Repeat chest radiograph showed improved pulmonary opacities bilaterally with residual atelectasis and improved gastric distension (Figure 3). The patient was discharged home the following day.

## Discussion

Our patient’s facial features such as Mallampati IV, short thyromental distance, and small chin suggested difficult intubation (Figure 1). Her other feature such as a large tongue relative to the oral cavity suggested potential obstructive breathing pattern under anesthesia and difficult mask ventilation. Her limited mouth opening would also make the insertion of the laryngeal mask airway extremely difficult. Our review of her previous anesthesia record indicated that she could be ventilated with a face mask with oral airway and that asleep nasal fiberoptic was successful. Hence, to prepare for this patient’s induction and intubation, we had appropriate oral and nasal airways available for mask ventilation, fiberoptic bronchoscope available and an anesthesiologist experienced in advanced airway management in the room. We also had the same equipment, oral and nasal airways and fiberoptic bronchoscope, and an experienced anesthesiologist available for extubation, which contributed to our prompt management of this patient’s airway complication.

Laryngospasm is an uncommon complication of anesthesia with an incidence of 0.87% in adults, 1.7% in pediatrics and 2.82% in infants [2,3]. The incidence is higher in children with obstructive lung disease, acute upper respiratory infection and passive cigarette smoke exposure [2]. It is often caused by a lack of inhibition of the glottis reflex due to an inadequate central nervous system suppression and provoked by stimuli such as extubation, bloody secretions, stimulation of the airway with either a laryngoscope or a suction catheter [1,2]. Laryngospasm is a condition associated with partial or complete closure of vocal cords. Laryngospasm with complete closure of the larynx presents as silent chest movements with no ventilation [2]. Our patient likely developed complete airway closure due to laryngospasm stimulated by the extubation and bloody secretions. Partial laryngospasm, also called partial glottic spasm, can present similar to upper airway obstruction, in which patients develop stridulous noises during inspiration and asynchronous movement of the chest and abdomen during respiration [2]. The conventional management of laryngospasm involves the use of CPAP with 100% oxygen to push open the vocal cords, the application of Larson Maneuver, administering a low dose (0.5-0.8mg/kg) IV propofol to deepen the depth of anesthesia, and administering 1-2mg/kg IV succinylcholine to relax the vocal cords if laryngospasm persists [2]. Larson Maneuver refers to the application of pressure on the styloid process behind the posterior ramus of the mandibles [2]. Succinylcholine is a reliable pharmacologic agent to break laryngospasm but is associated with adverse side effects such as bradycardia and arrhythmias [2]. Lorch and Sahn reviewed eight published reports of peri-intubation laryngospasm and suggested that the fact that some patients did not respond to succinylcholine may suggest unrecognized hypopharyngeal airway obstruction rather than laryngospasm as these patients had obese body habitus with short neck [4]. Our patient has facial features, such as a short neck and large tongue relative to the oral cavity, that make her prone to develop supraglottic airway obstruction. Our patient likely developed laryngospasm initially, which was relieved by propofol, and later developed supraglottic upper airway obstruction due to relaxation of supraglottic muscles by propofol. According to prior literature, a lower dose, such as 0.5-0.8mg/kg, of propofol or succinylcholine could be a more prudent choice to relieve the laryngospasm without increasing the risk of supraglottic airway obstruction. We created a table to illustrate the differences between true laryngospasm and supraglottic obstruction (Table 1). In Rajan’s letter to the editor, he commented that it is sometimes impossible to differentiate between true laryngospasm and unrelieved supraglottic obstruction and suggested that the diagnosis could be confirmed by directly visualizing the vocal cord using direct laryngoscope (DL), which at the same time, could relieve upper airway obstruction [5]. However, this suggestion could be difficult to be implemented in patients with difficult airways such as our patient. It is also important to ensure adequate depth of anesthesia prior to attempting DL as it can also stimulate another episode of laryngospasm during inadequate depth of anesthesia.

**Table 1:** Differences between true laryngospasm and supraglottic obstruction Abbreviations: ASA – American Society of Anesthesiologists physical status classification; OSA – obstructive sleep apnea.

	True Laryngospasm	Supraglottic Obstruction
Prevalence	Rare depending on surgical type, patient age, condition and anesthesia.	More common.
Patient Type	Young healthy patients with ASA 1 or 2.	Patient with risk factors for airway obstruction (i.e. OSA, obese body habitus, unusual facial features).
Presentation	Usually presents with high-pitch inspiratory stridor, but could also present with silent with chest movement and deteriorating oxygen saturation.	Usually presents with low-pitch snoring, but could present with silent chest movement and deteriorating oxygen saturation
Management	Usually unrelieved despite optimizing airway such as jaw lift, chin lift and airway adjuncts. Usually relieved with succinylcholine or deepening anesthesia.	Usually relieved with jaw lift, chin lift and airway adjuncts (oral airway, nasal airway, and laryngeal mask airway). Does not respond to succinylcholine.

**Figure 4:**
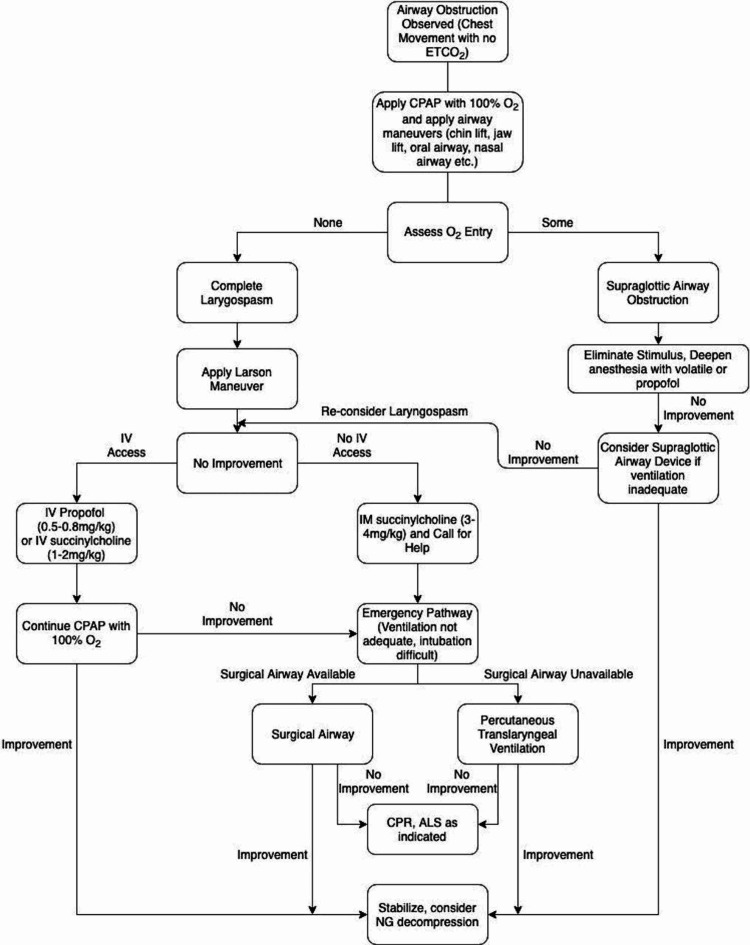
An algorithm for the management of laryngospasm and supraglottic obstruction in adult patients with difficult mask ventilation and difficult intubation. ETCO_2_ – End-tidal carbon dioxide; O_2_ - Oxygen; CPAP - Continuous positive airway pressure; IV - intravenous; IM - intramuscular; ALS - Advanced Life Support; CPR - Cardiopulmonary Resuscitation; NG - nasogastric [2,9].

Our patient has a rare genetic disorder called TCS, which has an estimated frequency of 1 in 50,000 live births [6]. The signs and symptoms include underdeveloped cheekbones, micrognathia, downward slanting eyes, absent, small or unusually formed ears [6]. These could lead to complications of respiratory problems, vision, cleft palate and hearing loss [6]. A retrospective study of 45 children in 2005-2009 with craniofacial deformities revealed that children with TCS had the most difficult airway management with DL view, often requiring multiple airway accessories and intubation techniques [7]. The two most common methods of airway management used were stylet and fiberoptic intubation [7]. Another retrospective study on TCS children showed that 53% of the 97 cases had modified Cormack Lehane (MCL) view grade 3 and grade 4 DL and failed intubation rate of 5% in 123 cases and that their MCL views become more difficult with increasing age [8]. Our patient served as an example who grew up with multiple oral and maxillofacial surgeries and developed an increasingly more difficult airway. We briefly attempted DL after the patient recovered from laryngospasm and had an MCL view of grade 4. We incorporated previous management of laryngospasm as well as the American Society of Anesthesiologists Difficult Airway Algorithm to propose an algorithm for management of laryngospasm and supraglottic obstruction in adult patients with difficult airway (Figure 4) [2,9]. In the current anesthesia practice, non-pediatric anesthesiologists will also encounter adult patients who have genetic disorders with craniofacial anomalies. It is thus important to recognize the challenges in managing laryngospasm in patients with congenital syndrome because mask ventilation may be difficult to achieve due to the facial anomalies, insertion of supraglottic airway device could be impossible due to distorted anatomy, and re-intubation could be difficult without advanced airway equipment and an emergent cricothyroidotomy may need to be performed to prevent hypoxic-ischemic encephalopathy. Re-intubation and cricothyroidotomy were considered in our case when the patient developed complete airway closure with laryngospasm, but not utilized because the patient quickly improved her oxygen saturation after propofol administration and positive pressure ventilation. At a tertiary medical center, it might be beneficial to assign a pediatric anesthesiologist to take care of these patients with genetic disorders with craniofacial anomalies. It is also important to have an anesthesiologist experienced in advanced airway management and a fiberoptic bronchoscope in close proximity to the patient during extubation of a patient with a difficult airway.

Several prevention strategies have been studied in order to reduce or eliminate laryngospasm. Patients’ oropharyngeal secretions should be thoroughly suctioned prior to extubation as bloody secretions could stimulate a laryngospasm response [2]. Complete reversal of neuromuscular blockade should be ensured to eliminate the risk of airway obstruction from residual neuromuscular blockade [10]. An opioid-sparing technique could be considered to minimize the risk of postoperative respiration depression [10]. A prospective multi-institutional observational study demonstrated that an increased number of laryngoscope attempts is associated with an increased incidence of laryngospasm [11]. A dose of 5mg dexamethasone could be used prior to extubation to reduce laryngeal edema from multiple intubation attempts [12]. The administration of magnesium (15-30mg/kg) prior to induction has been shown in a randomized trial to be associated with a decreased frequency of laryngospasm by deepening the anesthesia and enhancing muscle relaxation [13]. The use of 1-2mg/kg of lidocaine five minutes prior to tracheal extubation has been shown by a large meta-analysis in reducing pediatric laryngospasm [14]. A subhypnotic dose of 0.5mg/kg of propofol 60 seconds prior to extubation has been demonstrated to be highly effective in decreasing the incidence of laryngospasm [15]. A single dose of dexmedetomidine at 0.5µg/kg has been shown to be associated with a reduction in laryngospasm due to its attenuation of airway reflexes without prolonged recovery [16].

NPPE is an uncommon complication of anesthesia with an incidence of 0.1% and can occur as a complication of laryngospasm [4]. The pathogenesis of NPPE involves a high negative intrathoracic pressure, which causes a significant fluid shift from microvessels to perimicrovascular interstitium [17]. The inspiratory effort against a closed glottis, known as Mueller maneuver, increases the afterload, which in turn increases the pulmonary capillary hydrostatic pressure [17]. The disruption of alveolar epithelium and pulmonary microvascular membranes from severe mechanical stress also increases pulmonary capillary permeability and thus increase the fluid shift and then pulmonary edema develops [17]. The pulmonary edema fluid in post obstructive pulmonary edema is frequently blood-stained due to this disruption of the pulmonary capillaries. This is one of the features, which helps to distinguish post obstructive pulmonary edema from cardiogenic pulmonary edema. The most common risk factors for this complication are young age and healthy athletic males as their body types are capable of generating large negative intrathoracic pressures and therefore large hydrostatic pressure gradients [18]. Orthognathic surgery also placed patients at an increased risk of NPPE because the swelling of the oral cavities during such surgeries make the airway spaces narrower and increase the risk of upper airway obstruction whereas bleeding from the wounds could irritate the larynx and initiate laryngospasm [19]. It is important to keep NPPE in the differential in a patient with the aforementioned risk factors who recently developed laryngospasm or upper airway obstruction in order to initiate treatment timely. Laryngospasm-induced NPPE usually resolves in less than 36 hours [20]. The management of NPPE that does not spontaneously resolve includes non-invasive positive pressure ventilation or re-intubation. CPAP was not used in our case because our patient’s facial features will make a good seal with CPAP mask difficult. Other managements include diuretics and restriction of fluid [20].

## Conclusions

Through our case report, we hope to provide handy tools for future providers to manage true laryngospasm and NPPE in patients with difficult airways. It is important to keep in mind that emergent cricothyroid cannulation with oxygen insufflation or formal cricothyroidotomy may be needed to prevent hypoxic-ischemic encephalopathy. These patients need to be cared for in an environment where advanced airway equipment and staff who are skilled in the use of this equipment as well as in performing an emergent cricothyroidotomy are readily available.
